# Neurotrophins as Key Regulators of Cell Metabolism: Implications for Cholesterol Homeostasis

**DOI:** 10.3390/ijms22115692

**Published:** 2021-05-26

**Authors:** Mayra Colardo, Noemi Martella, Daniele Pensabene, Silvia Siteni, Sabrina Di Bartolomeo, Valentina Pallottini, Marco Segatto

**Affiliations:** 1 Department of Biosciences and Territory, University of Molise, Contrada Fonte Lappone, 86090 Pesche, Italy; m.colardo@studenti.unimol.it (M.C.); n.martella@studenti.unimol.it (N.M.); d.pensabene@studenti.unimol.it (D.P.); sabrina.dibartolomeo@unimol.it (S.D.B.); 2 Department of Cell Biology, UT Southwestern Medical Center, Dallas, TX 75390, USA; silvia.siteni@utsouthwestern.edu; 3 Department of Science, University Roma Tre, Viale Marconi 446, 00146 Rome, Italy; valentina.pallottini@uniroma3.it; 4 Neuroendocrinology Metabolism and Neuropharmacology Unit, IRCSS Fondazione Santa Lucia, Via del Fosso Fiorano 64, 00143 Rome, Italy

**Keywords:** BDNF, cholesterol, metabolism, neurotrophins, NGF, p75NTR, TrkA, TrkB

## Abstract

Neurotrophins constitute a family of growth factors initially characterized as predominant mediators of nervous system development, neuronal survival, regeneration and plasticity. Their biological activity is promoted by the binding of two different types of receptors, leading to the generation of multiple and variegated signaling cascades in the target cells. Increasing evidence indicates that neurotrophins are also emerging as crucial regulators of metabolic processes in both neuronal and non-neuronal cells. In this context, it has been reported that neurotrophins affect redox balance, autophagy, glucose homeostasis and energy expenditure. Additionally, the trophic support provided by these secreted factors may involve the regulation of cholesterol metabolism. In this review, we examine the neurotrophins’ signaling pathways and their effects on metabolism by critically discussing the most up-to-date information. In particular, we gather experimental evidence demonstrating the impact of these growth factors on cholesterol metabolism.

## 1. Introduction

At the end of the 19th century, the knowledge concerning neuroscience research underwent deep changes starting from the hypothesis that nerve cells need trophic support [1]. By a rare combination of intuition and scientific interpretation, this assumption was demonstrated by Rita Levi-Montalcini, Viktor Hamburger and Stanley Cohen in the early 1950′s with the discovery of nerve growth factor (NGF), the first growth factor characterized [2,3]. This was embodied in a conceptual postulate, known as neurotrophic theory, which sustains a crucial role of NGF and other neurotrophic factors in the regulation of neuronal survival, differentiation, and function [1]. 

Even though they were initially regarded as signaling molecules with specific tropism for neuronal cells, compelling evidence indicates that NGF and the other related factors, called neurotrophins (NTs), can promote the survival and the growth of several non-neuronal cells. Importantly, NTs affect a number of metabolic processes associated to the control of glucose levels, autophagy, oxidative stress, energy homeostasis and lipid metabolism [1,4]. During the last few years, experimental findings highlighted a role for NTs in cholesterol metabolism regulation. Cholesterol plays crucial roles in cell physiology, as it assures the proper functioning of cell membranes and regulates different intracellular molecular pathways involved in multiple biological processes [5]. Thus, it is not surprising to discover that signaling factors such as NTs, implicated in the growth, survival and differentiation of both neuronal and non-neuronal cells, could influence cell homeostasis by modulating, at least in part, cholesterol metabolism. 

The purpose of this review article is to collect and critically discuss the current knowledge about the role exerted by NTs in the regulation of cell metabolism, with particular emphasis on cholesterol metabolism. In addition, the most relevant pathways involved in NTs signaling and cholesterol homeostasis are also described, and integrated with the most up-to-date information.

## 2. Neurotrophins: From Cell Signaling to Metabolic Processes

NTs are a small family of growth factors significantly involved in the regulation, development and function of the nervous system [6,7]. In 1951, Nobel laureate Rita Levi-Montalcini discovered the nerve growth factor (NGF), a neutrophin able to enhance the growth of sensory and sympathetic neurons [8,9]. Later, other important NTs were discovered, as the brain-derived neurotrophic factor (BDNF), the neurotrophin-3 (NT-3) and neurotrophin-4 (NT-4) [10,11,12,13]. NTs are initially synthetized as pro-neurotrophins (pro-NTs), high molecular weight precursors of 240–260 amino acids. Pro-NTs are proteolytically cleaved by proteases to obtain mature neurotrophins with a molecular weight of approximately 13–14 kDa [14,15]. The production of mature NTs is cell-tissue dependent and their expression pattern may vary according to cell context and physiopathological condition [7]. For instance, proprotein convertase subtilisin/kexin 3 (PCSK9, or furin) is expressed in most mammalian cells, while proprotein convertase subtilisin/kexin 1 (PCSK1, or NEC1) and proprotein convertase subtilisin/kexin 2 (PCSK2, or NEC2) preferentially show neuronal expression. These enzymes expression is subjected to elegant physiological regulations, and a considerable amount of pro-NTs can be directly secreted in the extracellular milieu in specific contexts. Once secreted, mature NTs influence a variety of aspects related to the development and the functioning of the nervous system, such as neurite extension, axonal growth and neuronal differentiation, myelination, synaptic plasticity, survival and apoptosis [6,7,16]. In the extracellular space, pro-NTs may undergo proteolytic processing by the activity of plasmin and/or matrix metalloproteases (for extensive review, please see ref. [17,18,19]). Interestingly, it is now well-established that, independently from their mature forms, pro-NTs exert biological activity and may display different, even opposite, effects if compared to their mature counterparts [6,7]. 

### 2.1. Neurotrophin Receptors and Signal Transduction

**Figure 1:**
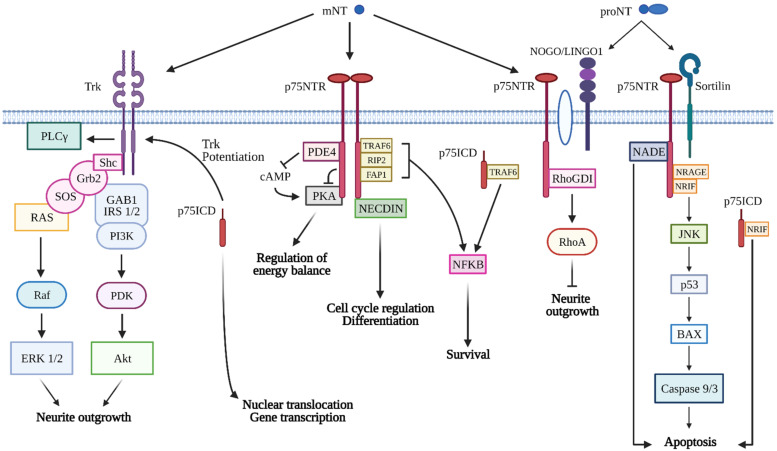
Signaling pathways mediated by neurotrophins’ binding to their receptors. Neurotrophins bind to two main receptor types: Trk receptors and p75NTR. Pro-neurotrophins (proNT) processing generates mature neurotrophins (mNT). Upon mNT stimulation, Trk receptors undergo autophosphorylation events, and recruit adaptor proteins responsible for the activation of MAPKs, PI3K and PLCγ signaling cascades, involved in several neuronal functions such as neurite outgrowth. mNT may also bind to p75NTR, stimulating its proteolysis which releases the intracellular domain (p75ICD) that is essential for signalling and for the regulation of gene transcription. p75NTR activation by mNT may promote neuronal survival by inducing NF-kB signaling. In addition, Necdin recruitment to p75NTR may regulate cell cycle progression and differentiation in a variety of cell types, whereas the interaction with PDE4 and PKA is involved in the regulation of energy balance. Importantly, in dependence on the cell context, p75NTR activation by mNT is able to potentiate the activation of Trk receptors. On the other hand, the binding of proNT to the p75NTR/Sortilin complex enhances the activation of cell-death-related pathways. When p75NTR interacts with NOGO/LINGO1, the recruitment of Rho GDI leads to the activation of RhoA, which in turn determines the inhibition of neurite outgrowth in neurons. FAP1, FAS-associated phosphatase 1; GAB1, GRB2 Associated Binding Protein 1; Grb2, growth factor receptor-bound protein 2; IRS 1/2, insulin receptor substrate 1/2; JNK, c-Jun N-terminal kinases; LINGO1, leucine-rich repeat and Immunoglobin-like domain-containing protein 1; NADE, p75NTR-associated cell death executor; p75NTR, p75 neurotrophin receptor; NRAGE, neurotrophin receptor-interacting MAGE homologue; NRIF, neurotrophin receptor-interacting factor; PDE4, phosphodiesterase 4; PDK, phosphoinositide-dependent kinase; PKA, protein kinase A; PLCγ phospholipase Cγ; RhoA, Ras homolog family member A; RIP2, receptor-interacting protein 2; Shc, SRC homology domain-containing protein; SOS, son of sevenless; TRAF6, TNF receptor-associated factor 6; Trk, tropomyosin receptor kinase. This figure is created with BioRender.

The cellular effects mediated by NTs and their precursors are strictly dependent on the binding of two different classes of membrane receptors: tropomyosin-receptor kinases (Trks) and the pan-neurotrophin receptor p75 (p75NTR) (Figure 1). Trks are transmembrane glycoproteins with an extracellular neurotrophin-binding domain comprising two cysteine-rich regions separated by a leucine-rich repeat, two tandem immunoglobulin G (IgG)-like domains located nearby the plasma membrane, a single-pass transmembrane region, and an intracellular domain with intrinsic tyrosine kinase activity. NTs bind the second IgG-like domain, resulting in Trk receptor homodimer formation, and subsequent triggering of tyrosine kinase activity by autophosphorylation [16]. Each neurotrophin selectively binds a different Trk: NGF activates TrkA; BDNF and NT-4 bind to TrkB; whereas NT-3 preferentially elicits TrkC activation [16,20]. However, Trks signaling is made more intricate by heterologous binding. For instance, it has been demonstrated that NT-3 and NT-4 can also bind to and activate TrkA, as well as NT-3, may induce the activation of TrkB [13,20]. The complexity of NTs signaling is also increased by the expression of Trks splicing variants, which have different properties if compared to the full-length (FL) receptors. In this context, several reports demonstrated the existence of Trk receptors with deletions in both the extracellular and intracellular domains [21,22,23]. These specific isoforms may act as dominant negative modulators, NTs sequestrants or trigger alternative signaling pathways when compared to FL receptors [24]. Experimental evidence highlighted that an 18 bp deletion in the extracellular domain alters TrkA activation by NT-3. In contrast, exon 9 deletion renders TrkB less prone to NT-4 and NT-3 stimulation [22,23]. TrkB.T1 is the most commonly expressed truncated isoform of TrkB [25,26] and it shows the same binding affinity to BDNF if compared to TrkB FL. However, its intracellular domain lacks the tyrosine kinase domain, resulting in dimerization inability and consequently, in the classic TrkB FL signal transduction pathways activation. TrkB.T1 is particularly expressed in the adult nervous system [25,27], predominantly in astrocytes [28,29,30]. The exact biological effects of TrkB.T1 signaling are not fully elucidated [31]. However, it has been observed that it may regulate intracellular calcium entry, as well as small Rho GTPase activity in astrocytes [28,32,33]. In their FL forms, the Trks act mainly through three major transduction pathways: phospholipase C-γ1 (PLC-γ1), Ras and phosphatidylinositol 3-kinase (PI3K) [34]. Trk phosphorylation at Tyr785 residue generally leads to the activation of the PLC-γ1 signaling pathway [24]. Indeed, PLC-γ1 catalyzes the hydrolysis of phosphatidylinositol 4,5-biphosphate to yield diacylglycerol (DAG) and inositol trisphosphate (IP3). DAG activates protein kinase C (PKC), whereas IP3 binds to its receptors and leads to the release of Ca^2+^ from the endoplasmic reticulum (ER) stores which, in turn, activates the Ca^2+^-calmodulin-dependent protein kinases. Several findings demonstrated that the involvement of the PLC-γ1 pathway in Trks activation is crucial for the induction and maintenance of synaptic plasticity [35]. In addition, PLC-γ1 influences key cellular processes, such as proliferation and differentiation, in both neuronal and non-neuronal cells [6,36,37,38,39,40]. The activation of Ras pathway requires the NT binding to Trk receptors, their phosphorylation at Tyr490 residue and the subsequent recruitment/phosphorylation of the adaptor protein Shc. Phosphorylated Shc binds to the Grb2-SOS complex, activating the guanine nucleotide exchange factor SOS. SOS removes GDP from the small GTPase Ras, which is now able to bind to GTP and switch to the active form. Subsequently, Raf kinase is activated by Ras, and in turn, phosphorylates ERK. ERK regulates the activation of several downstream substrates and mediates different cellular effects such as proliferation, differentiation, survival, cell growth and apoptosis [6,41]. Notably, ERK can enter the nucleus to regulate the activation of several transcription factors, including cAMP response element-binding protein (CREB), which controls transcriptional changes required for neuronal differentiation, neurite outgrowth and synaptic plasticity [42,43]. PI3K is activated upon recruitment operated by the adaptor complex Shc-Grb2-Gab1. Otherwise, it can also be activated by Ras. PI3K activity leads to the activating phosphorylation of Akt, a serine-threonine kinase crucially involved in cell survival and growth [6]. For instance, Akt suppressed BAD activity, inhibiting the promotion of signaling cascades involved in apoptosis [44]. 

Besides Trk receptors, NTs also induce their biological effects through p75NTR. P75NTR is a transmembrane glycoprotein receptor of approximately 75 kDa, belonging to the tumor necrosis factor (TNF) receptor family. P75NTR unselectively binds to all NTs with similar low affinity, whereas pro-NTs bind to p75NTR with high affinity [6]. The intracellular domain of p75NTR lacks catalytic activity, thus signaling pathways are mediated by the recruitment of adaptors and interacting proteins. p75NTR may form functional dimers, interacting with mature NTs. This interaction induces substantial conformational changes in the intracellular domain, leading to the recruitment of effector proteins [45]. TRAF6 is the most studied adaptor protein, and its activation is responsible for NF-kB-mediated survival [46,47]. NF-kB activation may also be elicited by RIP2 recruitment [48]. Importantly, it has been recently shown that p75NTR-RIP2 interaction facilitates the survival of cerebellar granule neurons by promoting NF-kB-dependent transcription of pro-survival genes [49,50]. p75NTR has also been reported to interact with Necdin. This MAGE-related protein together with E2F1, E2F4 and p53, represents a key regulator of the cell cycle, differentiation and survival in several tissues [7,51,52]. p75NTR activation is also implicated in the regulation of PKA signaling. Notably, phosphodiesterase PDE4A4/5 can be activated by p75NTR interaction, and this event leads to the increase of cAMP degradation and the subsequent PKA inactivation [53]. Furthermore, binding assays demonstrated that the intracellular domain of p75NTR interacts with both the catalytic and the regulatory PKA subunits. Hence, PKA activity may additionally be suppressed by direct interaction with p75NTR [54].

p75NTR not only forms homodimers, but also heterodimers with various receptors. These interactions frequently influence the binding to ligands and the consequent signaling pathways. For instance, pro-NTs have an increased affinity for p75NTR when receptor sortilin is co-expressed [55]. Pro-NTs-p75NTR–sortilin complex promotes the association of adaptor proteins involved in the induction of apoptotic cascades, including p75NTR-associated death executor (NADE), neurotrophin receptor-interacting factor (NRIF) and neurotrophin receptor-interacting MAGE homolog (NRAGE). These interactors mediate the stimulation of the c-Jun terminal kinases (JNK), which activate the transcription factor c-Jun and the tumor suppressor p53, resulting in the buildup of pro-apoptotic genes and, ultimately, cell death [47]. Specifically, it has been shown that p75NTR-dependent activation of p53 upregulates Bax expression and its mitochondrial translocation. These events result in cytochrome c release, caspase activation and neuronal apoptosis [56,57]. 

The formation of signaling complexes with Nogo and myelin-associated glycoprotein (MAG) facilitates the interaction of RhoGDI with the intracellular domain of p75NTR. Thus, p75NTR acts as a GDF displacement factor (GDF) that dissociates RhoA from RhoGDI. This event results in RhoA activation, which reflects in cytoskeleton rearrangement and inhibition of neurite outgrowth [58,59]. 

p75NTR is also often co-expressed with Trk receptors in diverse cell contexts. It has been reported that p75NTR interacts with TrkA [60], TrkB, and TrkC receptors [61], thereby modulating their affinity and response to NTs, and decreasing their endocytic internalization and degradation [62]. Notably, p75NTR potentiates the activation of Trk receptors, enhancing the survival and differentiation of neurons [63]. The molecular mechanisms by which p75NTR increases Trk activity are still not completely elucidate, and it was initially postulated that the two receptors may physically interact to form heterodimer complexes [60,61]. However, experimental findings demonstrated that there is no evidence for a direct interaction between p75NTR and TrkA extracellular domains, although the two receptors likely communicate through shared adaptor proteins or converging signaling pathways [64]. More recently, other studies highlighted that p75NTR and TrkA receptors are physically associated through the interaction of the intracellular domains, thus this complex can be formed independently of ligand stimulation [65]. 

Increasing evidence sustains that, similarly to Notch and amyloid precursor protein (APP), p75NTR is subjected to regulated intramembrane proteolysis, which generates soluble intracellular fragments capable of signaling properties. p75NTR is initially cleaved by the tumour necrosis factor-alpha converting enzyme (TACE/ADAM17) to yield a membrane-bound C-terminal fragment (CTF). Subsequently, CTF undergoes further cleavage by the γ-secretase, releasing the soluble intracellular domain (ICD) into the cytoplasmic compartment [66,67]. p75NTR ICD exerts several functions, ranging from survival to cell death, in relation to the co-receptors and/or the type of ligands [68]. In addition, recent reports indicated that p75NTR ICD may translocate to the nucleus, possibly regulating gene expression [7,68,69,70].

### 2.2. Neurotrophins in the Regulation of Cell Metabolism

Despite their critical role in the development, maintenance, survival and plasticity of the central nervous system, cumulative evidence indicates that NTs also as fundamental players in the regulation of metabolism and energy homeostasis. For this reason, these growth factors were designated as metabotrophins (from the greek *metabole*, and *trophe*, meaning “nutricious for metabolism”), highlighting the capability of NTs to regulate the activity of several cell processes, including those involved in glucose and lipid metabolism [71,72,73]. 

BDNF is the most extensively characterized NT concerning the control of energy homeostasis and mitochondrial metabolism. An interesting research study demonstrated that this growth factor affects energy metabolism in cortical developing neurons. BDNF upregulates the expression of the glucose transporter GLUT-3 and stimulates glucose utilization; a concurrent increase in amino acid uptake and protein synthesis are also observed upon BDNF administration: these events are essential to determine neuronal differentiation [74]. Signaling pathways mediated by BDNF through TrkB.T1/p75NTR interaction were shown to be determinants for the induction of glycogen synthesis in astrocytes. In contrast, when astrocytic p75NTR expression is reduced, the association between TrkB.T1 and adenosine receptor A_2A_R or dopamine receptor D1R is favored, leading to the activation of PKA and subsequent induction of glycogen hydrolysis [75]. BDNF influences glucose metabolism not only in brain cells but also in peripheral tissues: recent findings showed that it exerts opposite effects on the transcripts of phosphoenolpyruvate carboxykinase (Pepck) and glycogen synthase kinase 3b (GSK3b) in hepatocytes, suggesting a reduction in gluconeogenesis and a concurrent enhancement in glycogen synthesis [76]. The impact of BDNF on systemic glucose metabolism is sustained by its crucial role in controlling the secretion of pancreatic hormones. Notably, β-cells express the TrkB.T1 receptor, and stimulation with BDNF triggers the release of calcium from the intracellular stores, enhancing insulin secretion induced by glucose [77]. On the other hand, pancreatic α-cells express the TrkB receptor and are responsive to BDNF, since its administration significantly reduces glucagon secretion [78]. In this context, it appears that BDNF may exert a general hypoglycemic effect by coordinating insulin and glucagon release. Coherently with this notion, it is becoming increasingly clear that BDNF signaling may participate in the development of type 2 diabetes mellitus (T2DM) [79,80,81]: specifically, BDNF levels were found to be decreased in patients with T2DM, and a negative correlation between insulin sensitivity and BDNF plasma concentration was also observed [82,83]. Interestingly, similar effects were also noted in rodent experimental models, where BDNF was shown to restore the alterations in pancreatic insulin and glucagon content in obese diabetic mice. In addition, BDNF administration also improved insulin resistance, body temperature and oxygen consumption in diabetic mice [84]. Another interesting report suggests that BDNF actions on diabetic hyperglycemia can be mediated, at least in part, by the effects induced in the brain, since intracerebroventricular or intra-ventromedial hypothalamic nucleus BDNF administration significantly counteracts hyperglycemia in streptozotocin-treated rats [85]. Additionally, recent data sustain that BDNF may exert a protective role against endothelial cell dysfunction observed in diabetic vascular complications, by activating mitophagy and by attenuating mitochondrial alterations and oxidative stress [86].

Energy metabolism is also influenced by NGF. Neuronal differentiation induced by NGF is accompanied by changes in ATP and NADPH levels, as well as a rise in oxygen consumption in rat pheochromocytoma PC12 cells. NGF-mediated mitophagy is also activated to assure the clearance of exhausted mitochondria, whereas the concurrent induction of mitochondrial remodeling and biogenesis sustains the energetic needs required during neuronal differentiation [87]. Similar to BDNF, experimental findings highlight a role for NGF in the hormonal control of glucose metabolism. Increased glucose levels stimulate the release of NGF and its signaling by TrkA receptors expressed by pancreatic β-cells, which in turn promotes F-actin rearrangement, granule mobilization and insulin secretion [88]. Coherently with its hypoglycemic effect, NGF efficiently ameliorates the molecular alterations reminiscent of insulin resistance in basal forebrain cholinergic neurons [89]. In addition, NGF administration counteracts ER stress and apoptosis observed in Schwann cells during diabetic peripheral neuropathy, and efficiently attenuates neuronal death under glucose fluctuation in diabetic encephalopathy [90,91]. In contrast to BDNF and NGF, very little experimental evidence highlights the involvement of NT-3 or NT-4 in the regulation of energy homeostasis. However, NT-3 may exert important modulatory effects on mitochondrial dynamics increasing mitochondrial density along embryonic sensory axons [92]. The effects of NT-3 on cell energy metabolism were also studied in the pathological context of diabetes. Chiefly, NT-3 prevents the depolarization of the mitochondrial inner membrane in sensory neurons of streptozotocin-treated mice [93], and efficiently counteracts the elevation of blood glucose levels observed in obese diabetic mice [94]. The metabotrophic activity of NTs is further corroborated by their modulatory role on skeletal muscle. Importantly, BDNF is expressed in skeletal muscle, and reprograms fiber metabolism by inducing a glycolytic phenotype [95]. Similar results were recently highlighted for NGF signaling: cell culture and in vivo experiments demonstrated that both satellite-cell-derived myoblasts and adult skeletal muscles express appreciable levels of p75NTR. Interestingly, skeletal muscle cells also synthetize NGF, with proNGF being the most predominant form. In this context, it has been reported that proNGF/p75NTR axis promotes the slow-to-fast fiber type transition by suppressing the slow/oxidative program, and by facilitating the induction of fast/glycolytic markers [7].

The regulation of cell metabolism by NTs is extended to the modulation of autophagy. Autophagy is a highly conserved biological process that is generally induced in response to nutrient starvation. It also mediates the degradation of macromolecules, and promotes the clearance of damaged organelles via the lysosomal pathway [96]. Under physiological conditions, basal autophagy flux is essential to maintain cellular homeostasis by participating in the routine turnover of cellular trash. Under stress and nutrient-deprivation, autophagy sustains cell survival by regenerating metabolites for the energy supply required during cell growth. Conversely, under pathological conditions, excessive induction of autophagy may be detrimental, leading to self-digestion and the degradation of key cellular components [96,97]. Most literature data point out a negative regulation on autophagy by BDNF [4]. For instance, BDNF signaling blocks autophagy in the forebrain of adult mice, and this event promotes synapse plasticity and memory [98]. Blockade of autophagy flux is also essential to guarantee the BDNF-mediated neuroprotective effects against mitochondrial dysfunction [96]. The mechanism by which BDNF suppresses autophagy seems to be ascribable to the activation of the TrkB signaling pathway. Specifically, it has been well-established that TrkB activation by BDNF elicits PI3K/akt/mTOR pathway, which is one of the most critical negative regulators of autophagy induction [15]. Similar to what was observed for BDNF, recent findings show that both NT-3 and NT-4 acts as autophagy inhibitors in different physiopathological conditions, such as colorectal cancers and functional recovery after spinal cord injury [99,100]. On the other hand, contradictory information is available concerning the involvement of NGF in autophagy modulation. Whereas some reports highlight that NGF inhibits autophagy [101,102], others provide evidence for NGF-mediated autophagy activation [103,104]. The nature of these conflicting results is not clear, however, it can be speculated that NGF may exert opposite roles on autophagy modulation, in dependence on the physiopathological context, the nutrient status of the cell, the relative abundance and the concurrent expression of TrkA/p75NTR receptors.

It has been well established that NTs may act as potent regulators of redox balance. NGF, BDNF and NT-4 exert neuroprotective effects by attenuating oxidative stress [105]. The antioxidant activity induced by these growth factors confers resistance to neuronal damage during neurodegenerative conditions, and is associated with the promotion of Nrf2 nuclear translocation and the subsequent heme oxygenase 1 (HO-1) upregulation [106,107,108,109]. The link between NTs and oxidative stress is further highlighted by the fact that drinking habits and inadequate eating regimens can disrupt NTs metabolism, possibly by affecting ROS production. For instance, perinatal alcohol exposure, as well as chronic alcohol consumption during adulthood, is often associated with increased oxidative stress and altered NTs levels [110,111,112]. Interestingly, it has been observed that not only maternal but also paternal alcohol exposure can significantly impact NGF and BDNF levels in the brains of male offspring [113]. In addition, several lines of evidence indicate that polyphenols, powerful antioxidant compounds able to counteract oxidative damage induced by alcohol exposure, may elicit changes in NGF and BDNF expression as well as in their receptors [114,115,116,117,118].

Few studies suggest that NTs can also govern cell lipid metabolism. As an example, NGF-induced neuronal differentiation of PC12 cells is paralleled by changes in lipid metabolism [119]. Additionally, exercise can induce BDNF expression in skeletal muscle cells, which acts as a myokine to enhance fatty acid oxidation through AMPK activity [120]. Ultimately, p75NTR-influencing lipolysis and fat oxidation in adipocytes additionally support a part for NTs in the management of lipid homeostasis [54].

## 3. Neurotrophins and Cholesterol Metabolism

Increasing experimental evidence demonstrated that NTs exert important modulatory roles in several metabolic processes, and raised basic questions about their prospective involvement in the regulation of cholesterol metabolism. In this section, we will summarize and update the most outstanding discoveries underlying the complexity of cholesterol regulation in the whole body as well as in the brain compartment. Additionally, we will review the current knowledge on how NTs signalling may influence cholesterol homeostasis.

### 3.1. Overview of Cholesterol Homeostasis

Cholesterol is the most abundant sterol in animal tissues, and its homeostasis is essential for cell functioning and metabolism. It is mainly located on cell membranes, where it contributes to regulating the fluidity and the permeability of the phospholipidic bilayer; it is also involved in the modulation of membrane architecture and vesicle trafficking. Furthermore, cholesterol participates in the formation of specialized membrane microdomains known as lipid rafts [121,122]. 

In addition to its functions on the cell membrane, cholesterol is the precursor of bile acids, steroid hormones and vitamin D [5,123]. Alterations in cholesterol homeostasis areat the root of various congenital human diseases such as Niemann–Pick type C disease (NPC) [124,125] and Smith–Lemli–Opitz syndrome (SLOS) [126]. Additionally, increasing evidence suggests a tight relationship between alterations in cholesterol metabolism and different pathological conditions including many types of cancer [127,128,129,130,131], and neurodegenerative diseases such as Alzheimer’s disease (AD) [132,133,134], although the establishment of a direct connection is still unclear [135]. 

Proper intracellular cholesterol levels are maintained by the fine-tuning of de novo biosynthesis, uptake, efflux, and accumulation. From a systemic point of view, the regulation of cholesterol levels relies upon the interplay among several tissues and organs, which ensure homeostasis maintenance [136]. 

De novo synthesis is assured by the mevalonate (MVA) pathway which, starting from the acetate, leads to the production of cholesterol through ~ 30 enzymatic reactions. In humans, most cells can biosynthesize cholesterol. Notably, this biosynthetic pathway is particularly active in the intestines, muscles, and skin cells [137]. Nevertheless, about 50% of the total synthesis occurring in the whole body takes place in hepatocytes [138]. 

One of the most relevant proteins of this multi-enzymatic process is the 3β-hydroxy-3β-methylglutaryl CoA reductase (HMGCR). This Endoplasmic Reticulum (ER)-resident enzyme reduces the 3β-hydroxy-3β-methylglutaryl CoA (HMG-CoA) to MVA. HMGCR represents the key and rate-limiting enzyme for cholesterol biosynthesis, thus its expression and activity are finely regulated at transcriptional, translational and post-translational levels [139]. 

Like other proteins involved in cholesterol homeostasis, HMGCR contains a sterol-sensing domain (SSD), which confers sensitivity to sterol levels in the ER membrane. In fact, when sterol levels are high, HMGCR interacts with the ER-resident protein insulin-induced gene (INSIG), thus undergoing ubiquitylation and subsequent proteasomal degradation [140]. 

Besides HMGCR degradation, long-term regulation of cholesterol metabolism is also operated by sterol regulatory element-binding proteins (SREBPs) transcription factors. Specifically, SREBP2 selectively regulates genes involved in cholesterol homeostasis [141,142]. When intracellular cholesterol levels decrease, SREBP2 is able to recognize and bind to specific DNA sequences called sterol regulatory elements (SREs), eliciting the transcription of several genes involved in cholesterol biosynthesis and uptake, including *hmgcr*, squalene monooxygenase (*sqle*) and low-density lipoprotein receptor (*ldlr*) [143]. 

SREBP2 is localized on the ER membrane, and its activation requires its transferring to the Golgi network. This event is mediated by the binding to SREBP cleavage activating proteins (SCAPs), which act as sterol sensors [144]. 

In the condition of sterol depletion, the SCAP/SREBP complex exits the ER and reaches the Golgi network, where SREBP undergoes proteolytic activation. Then, SREBP-derived N-terminal fragments are transcriptionally active and enter the nucleus to upregulate the expression of target genes [142]. Conversely, as intracellular sterol content rises, the SCAP/SREBP complex is restrained in the ER due to the interaction with INSIG proteins, thus preventing SREBP proteolysis and the subsequent gene transcription [144]. Even though the regulation of the SREBP pathway is well characterized, several modulatory mechanisms still remain poorly elucidated, and ongoing researches continuously shed light on novel players of cholesterol homeostasis. Four independent research groups recently demonstrated that the uncharacterized gene *C12orf49* is critically involved in the transcriptional control of cholesterol metabolism. Notably, *C12orf49* codifies for a glycosylated Golgi-resident membrane protein that is responsible for the proper SCAP expression and localization to the Golgi compartment. In addition, it has been demonstrated that site 1 protease (S1P) cleavage activity is promoted by the interaction with C12orf49, which in turn reflects in the proteolytic processing of several substrates, including SREBP [145,146,147,148].

Increasing evidence highlights that cholesterol biosynthesis is deeply subjected to epigenetic control [149]. For instance, very recent findings reported that the chromatin remodeling protein BRG1 is directly recruited to the SCAP promoter, influencing its transcription and the consequent SREBP maturation in liver cells [150]. The euchromatic histone-lysine N-methyltransferase 2 (EHMT2) acts as a cholesterol biosynthesis suppressor. EHMT2 inhibition by BIX01294 induces the expression of *srebf2* (encoding for SREBP2) through the reduction of H3K9me1 and H3K9me2 at the promoter [151]. Other reports also revealed that cholesterol biosynthesis may be strongly affected by the activity of bromo- and extra-terminal domain (BET) proteins, namely BRD2, BRD3, BRD4 and BRDT. Specifically, ChIP–seq and ChIP–qPCR analysis provided evidence that BRD4 is recruited to the promoters of a specific subset of genes, whose products belong to cholesterol biosynthetic pathway, such as farnesyl-diphosphate farnesyltransferase 1 (FDFT1), 24-dehydrocholesterol reductase (DHCR24), 7-dehydrocholesterol reductase (DHCR7) and mevalonate diphosphate decarboxylase (MVD). Importantly, BRD4 suppression by genetic knockdown or pharmacological inhibition efficiently displaces BRD4 from the promoters, thus reducing the expression of these target genes [152]. The epigenetic regulation of cholesterol biosynthesis by BET proteins is further sustained by another recent evidence, showing that BRD2 is recruited to the sigma 2 receptor (*S2R*), and cooperates with SREBP2 to regulate the subsequent expression of downstream targets involved in cholesterol metabolism [153]. Furthermore, BET inhibition by JQ1 significantly reduces the expression of cholesterologenic proteins, such as SREBP2 and HMGCR, which reflects in a decreased intracellular cholesterol content [154].

Short-term regulation of cholesterol biosynthesis is mainly achieved by post-translational events that regulate HMGCR activity. Notably, the phosphorylation of a serine residue near the catalytic domain (Ser872 in humans) by AMP-activated protein kinase (AMPK) inhibits HMGCR activity. On the other hand, the dephosphorylation of the same residue, exerted by the protein phosphatase 2A (PP2A), reactivates the enzyme [155]. 

Even though an important source of cholesterol comes from endogenous biosynthesis, cholesterol intake from food sources represents about 22% of total plasma cholesterol [156]. Cholesterol absorption occurs on the apical surface of enterocytes in the intestinal lumen and on the bile canalicular membrane of hepatocytes, where the Niemann–Pick type C1-Like 1 protein (NPC1L1) is expressed [157]. Specifically, cholesterol interacts with the N-terminal domain of NPC1L1 [158]. Differently, the C-terminal domain presents an endocytic signal sequence YVNxxF to allow the internalization of NPC1L1; whereas a QKR sequence interacting with LIM domain and actin-binding protein 1 (LIMA1), is involved in the modulation of NPC1L1 trafficking [159,160]. NPC1L1 is localized in the endocytic recycling compartment (ERC) but, upon cholesterol depletion, the protein is exposed to the cell membrane [161,162]. Cholesterol binding to NPC1L1 leads to the dissociation of the NPC1L1 C-terminal tail from the cell membrane. This conformational change makes the endocytic motif accessible for the NUMB endocytic adaptor protein. NUMB activates the clathrin-mediated endocytosis by recruiting clathrin on invaginated microdomains, then the endocytic vesicles are internalized [159]. Subsequently, vesicles move along the actin filaments up to the ERC and NPC1L1 is recycled to the cell membrane for reuse [160,161,163,164].

Another well-known player involved in cholesterol metabolism is the low-density lipoprotein (LDL) receptor (LDLr), which mediates the uptake of cholesterol from the bloodstream [165]. This cell surface glycoprotein mediates the LDL endocytosis via its extracellular ligand-binding domain [166,167]. Cellular lipid uptake from LDL is ensured by the presence of apolipoproteins such as apoE and apoB100, which are incorporated into very-low-density lipoprotein (VLDL) and LDL, and are responsible for the binding to LDLr. Subsequently, the interaction of these proteins with their receptor leads to membrane invagination and endocytosis [168]. Due to the acid endosomal compartment, LDLr undergoes a conformational change and dissociates from LDL, and is eventually recycled back to the cell surface to start a new round of endocytosis [169,170,171]. 

Cholesteryl esters contained in LDL are hydrolyzed by lysosomal acid lipase to yield free cholesterol, which is exported from the lysosomal lumen and delivered to other cellular compartments via NPC1 and NPC2 [172,173]. Notably, mutations occurring on *npc1* or *npc2* genes determine an aberrant buildup of cholesterol into the lysosomal compartment, leading to the occurrence of a lysosomal lipid storage disorder known as NPC [174]. 

As already aforementioned, almost all mammalian cells can synthetize cholesterol. However, plenty of them are unable to efficiently catabolize it. Thus, cholesterol excess can leave the cell through efflux, or can be stored as cholesteryl-ester droplets. 

Several proteins are involved in the efflux of cholesterol, such as ATP-binding cassette (ABC) subfamilies A and G [136] and extracellular apolipoproteins, which function as cholesterol acceptors [156]. ABCG5 and ABCG8 are almost exclusively expressed on the apical surface of hepatocytes and enterocytes: in this context, these transporters are involved in the excretion of cholesterol into the bile and the intestinal lumen, so that it can be eliminated by faeces [175].

The extracellular efflux does not constitute the only fashion through which mammalian cells can regulate the excessive concentration of intracellular cholesterol. Indeed, the production of cholesteryl esters, together with their storage in lipid droplets, represents another pivotal pathway that prevents free cholesterol accumulation. The Acyl-coenzymeA:Cholesterol Acyl-Transferase 1 (ACAT1) and ACAT2 isoenzymes are integral membrane proteins that take part in the esterification of cholesterol in different cell types [176,177]. ACAT2 is mostly expressed in enterocytes and hepatocytes, and can be activated by the presence of the cholesterol itself to catalyze the esterification of different sterols or steroids containing a 3β-hydroxyl group with fatty Acyl-CoA [178,179,180]. The experimental data highlight that ACAT2 is more efficient in esterifying 25-hydroxycholesterol and derivatives from bile acids, but less efficient in esterifying cholesterol itself when compared to ACAT1 [181]. Interestingly, it has been demonstrated that ACAT2 activity is strongly associated with oxidative stress. In particular, the accumulation of sterol and fatty acids induces the production of reactive oxygen species (ROS) which oxidize ACAT2 at cysteine residue (Cys277), thus promoting the stabilization of the protein and enhancing storage and/or export of cholesterol [136].

### 3.2. Cholesterol Metabolism in the Brain

The central nervous system (CNS) represents the richest organ in cholesterol of the whole body, as almost 20% of this sterol is located in the brain [182]. The involvement of cholesterol in the CNS has been well established in many physiological processes by a plethora of studies. About 70–80% of the cholesterol in the adult brain can be partially found in the plasma membrane of neurons and astrocytes, where it influences cellular morphology, stabilizes cell surface receptors, and modulates synaptic transmission. However, the majority of this molecule is localized in myelin sheaths produced by oligodendrocytes, devoted to the insulation of the axons required for the saltatory impulse of the action potential [183]. Despite these roles, cholesterol is more than a simple structural component. Cholesterol is needed to form new membranes for axons or dendrites and for the development of pre-/postsynaptic spines [184,185,186]. Furthermore, it is also involved in axonal guidance regulation [187], synaptogenesis [188], microtubular transport of synaptic vesicles through the cytosol [189], and neurotransmitter release [168]. Cholesterol is also a pivotal constituent of lipid rafts, and its presence regulates the activity of membrane receptors such as γ-aminobutyric acid (GABA) receptor [190,191,192] or neurotrophin receptors (Trk and p75NTR) [193]. Considering its crucial role in the homeostasis maintenance in brain cells, it should not surprise that alterations of cholesterol metabolism are associated with a number of pathological conditions in the CNS. As a matter of fact, several neurodegenerative diseases and neurodevelopmental disorders are linked to alterations of cholesterol homeostasis, such as AD and Parkinson’s disease (PD), NPC, Huntington’s disease (HD) and Autism Spectrum Disorders (ASD) among others [194,195,196,197,198,199,200].

**Figure 2:**
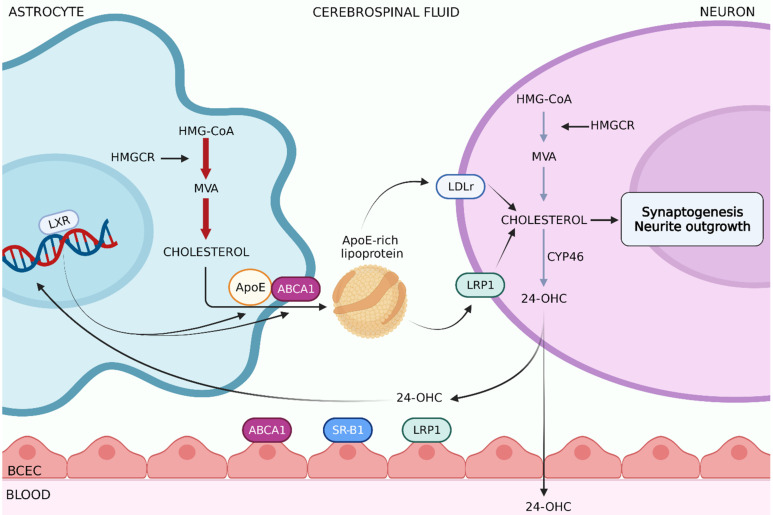
Cholesterol metabolism in the CNS. Cholesterol in the brain is assured by de novo biosynthesis requiring a multistep pathway. HMGCR is responsible for the conversion of HMG-CoA in MVA and represents the key and rate-limiting step. In the adult CNS, neurons reduce their own cholesterol synthesis and import this lipid from astrocytes, which secrete apoE rich-lipoproteins through ABCA1 transporters. ABCA1 and apoE transcription is elicited by LXR, whose activity is modulated by 24-OHC. Cholesterol uptake in neurons is ensured by LDLr and LRP1, particularly expressed on the cell membranes of neurons. Cholesterol excretion in the brain is mainly promoted by its conversion to 24-OHC catalyzed by CYP46. Abbreviations: 24-OHC, 24(S)-hydroxycholesterol; ABCA1, ATP-binding cassette transporter A1; apoE, apolipoprotein E; BCEC, brain capillary endothelial cells; CYP46, cholesterol 24-hydroxylase; HMGCR, 3-hydroxy-3-methylglutaryl-CoA reductase; LDLR, LDL receptor; LRP1, LDLr-related protein 1; LXR, liver X receptor; MVA, mevalonate; SR-B1, scavenger receptor class B member 1. This figure is created with BioRender.

The blood–brain barrier (BBB) efficiently prevents the uptake of lipoproteins from the systemic circulation. Thus, brain cholesterol is produced by means of de novo synthesis *in situ,* which is finely regulated in diverse brain cells in dependence on the developmental stage [201]. It is widely accepted that during the embryogenesis—before astrocyte’s differentiation—neurons autonomously synthetize cholesterol through the Kandutsch–Russel pathway [202,203]. However, during the postnatal period, neurons decrease or even interrupt their own synthesis whereas cholesterol production becomes particularly lively in astrocytes, through the Bloch Pathway [182,203,204]. Taken together, this evidence strengthens the hypothesis that mature neurons reduce or even abandon cholesterol synthesis to save the energy committed to the generation of the action potentials, thus relying on astrocytes to satisfy their cholesterol needs (Figure 2). Notably, experimental studies have highlighted that mice lacking cholesterol synthesis in adult neurons were phenotypically identical to controls, pointing out how cholesterol synthesis is not a crucial process in adult neurons [205]. In other words, the shutdown of cholesterol synthesis, counterbalanced by the increase in the intercellular trafficking of cholesterol from astrocytes via apoE-rich lipoproteins, ensures the ability to preserve energy in adult neurons for electrical activity. The export process begins in astrocytes, where newly-synthetized cholesterol is transferred by ABCA1 to extracellular lipid-free apoE [206]. Together with *apoE*, the *abca1* gene is expressed through the activation of Nuclear Liver X Receptors (LXRs). These transcriptional regulators sense the rising amount of cholesterol metabolites, such as cytosolic 24(S)-hydroxycholesterol (24-OHC) [182]. Through a feed-forward mechanism, LXRs interact with 24-OHC and other oxysterols inducing the expression of the aforementioned genes [207]. 

The uptake of apoE-rich lipoproteins in neurons is then mediated by endocytosis via LDLr family members, i.e., LDLr and LDLr-related protein 1 (LRP1) [202]. In vitro studies have shown cholesterol in apoE-rich particles to increase the synaptic responses by improving presynaptic functions and dendrite differentiation [208,209]. Coherently with the neurotrophic effect of the intercellular cholesterol transport, LDLr or apoE deficiency severely impacts adult hippocampal neurogenesis [210,211]. ApoE is mainly produced by astrocytes, even though neurons are able to express apoE as well, in case of brain injuries [212]. Recently, it has been discovered that astrocyte-derived apoE increases histone acetylation in neurons, enhancing the transcription of immediate early genes involved in memory consolidation [213]. In humans, apoE is polymorphic, meaning it is present in three different codifying alleles: *apoE2, apoE3* and *apoE4*. ApoE4 polymorphism is one of the most well-studied examples of how cholesterol metabolism is linked to neurodegeneration. Specifically, apoE4 is likely to be involved in late-onset Alzheimer’s disease, and is the most common risk-factor identified up to now for this pathology [214]: it affects amyloid-β aggregation and clearance [215,216], elicits synapse loss [217], and leads to the activation of microglia and astrocytes, thus causing neuroinflammation [218,219,220]. 

Once cholesterol-rich lipoproteins are endocytosed by the interaction between apoE and lipoprotein receptors, the apoE/cholesterol/LDLr complex is hydrolyzed, and free cholesterol is released and distributed through the different intracellular compartments [221] via NPC1 and NPC2, both expressed in neurons and glial cells [222]. 

As established before, due to its involvement in key neuronal processes, the amount of cholesterol in the brain must be finely controlled to ensure the correct brain functioning. Indeed, the presence of storage or secretion mechanisms is essential to counteract the putative excess of this sterol.

For instance, cholesterol in excess is esterified by ACATs enzymes mainly in the ER and stored in lipid droplets, representing about 1% of the total cholesterol content in the brain [223]. Despite ACATs are expressed in both neurons and glial cells, their activity is higher in neurons, where the esterification process strongly contributes to the maintenance of proper free cholesterol levels [224]. 

Besides esterification, the formation of oxysterols by hydroxylases represents the main way for cholesterol excretion from the brain. Cholesterol 24-hydroxylase is selectively expressed in the CNS, and is responsible for the conversion of cholesterol into its primary brain metabolite, 24-OHC: notably, 40% of cholesterol is released from the brain as 24-OHC [225]. This enzymatic conversion occurs in both neurons and glia cells, even though the major expression of cholesterol 24-hydroxylase takes place in neurons, especially in the pyramidal cells of the cortex and Purkinje cells of the cerebellum [226,227,228]. Oxysterols flow from the brain to the systemic circulation as a result of their capability to efficiently cross the BBB [229,230]. 

Another mechanism for cholesterol excretion is based on ABC transporters. In particular, ABCA1, ABCG1 and ABCG4 isoforms mediate cholesterol efflux from neurons to lipid-free apoA1 and High-Density Lipoprotein (HDL) particles [231]. Subsequently, secreted cholesterol-rich particles are released into the cerebrospinal fluid [232,233,234]. These lipoproteins also enter the systemic bloodstream by interacting with specific receptors such as LRP1 and scavenger receptor class B type I (SR-BI), both expressed in endothelial cells of brain capillaries [235,236]. 

Taken together, literature data highlight that cholesterol metabolism in the brain is controlled by intricate homeostatic mechanisms. In this context, neurons autonomously produce cholesterol during embryogenesis. Conversely, intercellular transport from astrocytes assures to neurons their cholesterol needs in the adult brain. Despite an adequate amount of cholesterol is essential to guarantee the proper activation of several neuronal processes, too much cholesterol may be detrimental. Thus, neurons manage the excess of cholesterol through different systems: the esterification, involving intracellular storage, or the excretion, using specific transporters. In addition, the conversion of cholesterol into more lipophilic molecules able to cross cell membranes (especially in the case of BBB), represents an important point of regulation to ensure that surplus cholesterol may efficiently exit the brain.

### 3.3. Regulation of Cholesterol Metabolism by Neurotrophins

As aforementioned, both cholesterol metabolism and neurotrophin pathways have been widely described as fundamental processes for the proper functioning of the whole body, including the CNS. A plethora of experimental evidence highlights that the presence of cholesterol in lipid rafts is essential to assure the correct translocation of neurotrophins’ receptors on the plasma membrane, and its depletion disrupts the receptor functions and the downstream signaling cascades [237,238]. On the other hand, the role of NTs in the modulation of cholesterol metabolism is still elusive and deserves further investigation. In this section, we will summarize the current knowledge proposing these growth factors as master regulators of cholesterol homeostasis (Table 1).

As previously reported, NGF is involved in the modulation of lipid metabolism. PC12 is a pheochromocytoma cell line derived from the peripheral nervous system (PNS) and it is able to assume a neuronal-like phenotype following appropriate stimuli. It has been observed that, upon NGF stimulation, these cells stop proliferating and undergo neurite extension. These evident morphological changes are accompanied by an increase in the small isoprenylated and methylated GTPases, highlighting the importance of protein prenylation in the acquisition of the neuronal-like phenotype induced by NGF. HMGCR activity is not only required for cholesterol production, but also for the synthesis of prenylated moieties employed for the post-translational modification and the subsequent attachment of small GTPases to cell membranes. In this context lovastatin, a powerful HMGCR inhibitor, reduces the amount of membrane-bound small GTPases, sustaining that NGF leads to an increase in the activity of the rate-limiting enzyme of the cholesterol biosynthetic pathway [239].

In addition to HMGCR, it has been reported that NGF may modulate the expression levels of other proteins involved in cholesterol metabolism, such as LRP1 and LDLr [240,241,242].

Following NGF administration, LRP1 membrane exposure and mRNA levels rapidly increase in GT1–1 Trk and PC12 cell lines. Interestingly, NGF treatment elicits the phosphorylation of the LRP1 cytoplasmic tail, and gives rise to the LRP1 endocytic activity [240]. More recently, NGF was also shown to activate the LRP1 promoter, further corroborating that NGF-mediated regulation of LRP1 occurs, at least in part, at the transcriptional level [241]. Taken together, these results confirm that NGF governs both short- and long-term regulation of LRP1 simultaneously with the neuronal differentiation process [240]. 

Other reports underlined that NGF increases LDLr expression levels in the pheochromocytoma cell line PC6.3 and in cultured septal neurons from embryonic rat brain, in a dose-dependent manner. The mature form of this neurotrophin induces *ldlr* gene transcription mainly through interaction with the TrkA receptor. LDLr upregulation is also promoted by pro-NGF binding to p75NTR receptor: coherently, it has been shown that p75NTR blockade selectively prevents the pro-NGF-induced increase in LDLr, whereas the upregulation of this receptor by mature NGF was suppressed upon TrkA inhibition. Similar to pro-NGF, pro-BDNF binding to p75NTR is also capable to enhance LDLr expression in PC6.3. Therefore, these data indicate that both mature NGF and pro-NGF influence LDLr expression by activating TrkA and p75NTR, respectively. In addition, the administration of both NGF and pro-NGF intensifies the uptake of lipoprotein particles. From a functional point of view, it has been revealed that the increased lipoprotein uptake promoted by NGF is a crucial mechanism that reinforces neurite outgrowth [242]. The effect of NGF on lipoprotein receptors is not delimited to LRP1 and LDLr, but also extends to apoER2. Differently from other LDLr family members, apoER2 only binds apoE-containing lipoproteins, and presents a high affinity for unrelated ligands such as reelin and clusterin. Besides mediation of macromolecules via endocytosis, apoER2 undergoes proteolytic cleavage and binds adapter proteins to activate different signal transduction pathways, most of them involved in brain development [243]. In this context, literature data elegantly illustrated that NGF triggers apoER2 proteolytic processing through TrkA-mediated signaling pathways [244]. NGF also influences cholesterol uptake by affecting the expression of apolipoproteins. For instance, it has been found that NGF treatment increases apoE transcript in PC12 cells, and this induction is attenuated by MAP kinase (MAPK) and PKC inhibitors, indicating that NGF-induced transcription of apoE requires the activation of TrkA/MAPK and TrkA/PKC axis [245].

Besides NGF, other experimental works pointed out a role for BDNF in the regulation of cholesterol metabolism. The results obtained by Suzuki and colleagues show that BDNF, by interacting with the TrkB receptor, leads to a selective up-regulation of *hmgcr* and mevalonate pyrophosphate decarboxylase (*mpd*) transcripts in neurons, but not in glial cells [188]. This evidence was also confirmed by inhibiting HMGCR with mevastatin and squalene synthase with zaragozic acid, which efficiently prevents the BDNF-mediated increase in cholesterol biosynthesis. Interestingly, cholesterol enrichment after BDNF treatment is particularly visible in neuronal lipid rafts, and was paralleled by a concurrent increase in the expression of presynaptic proteins. In addition, electrophysiological approaches demonstrated that BDNF-mediated de novo cholesterol synthesis is involved in the generation of a readily releasable pool of synaptic vesicles [188]. 

Other conclusions can be drawn from the results collected by Spagnuolo and collaborators, who recently highlighted the ability of BDNF to regulate apoE biosynthesis and the efflux of cholesterol from astrocytes, as well as its incorporation into neurons [30]. Specifically, BDNF stimulates cholesterol secretion from normal human astrocytes (NHA) and glioblastoma cell line U87 MG and, concurrently, increases ABCA1 expression levels. These events are also accompanied by an increase in Erk1/2 phosphorylation [30]. It has been well-established that TrkB activation by BDNF upregulates the Erk1/2 signaling pathway [251] and regulates ABCA1 expression in different cell types [252,253]. Furthermore, the Erk1/2 pathway is involved in the increase of apoE expression and secretion by astrocytes [254]. Hence, these data support the hypothesis that BDNF-induced Erk1/2 activation may play a role in the modulation of apoE and ABCA1 in NHA and U87 MG glial cells. 

In addition to cholesterol efflux from astrocytes, the authors revealed that BDNF administration increases *hmgcr* expression at mRNA level in the neuron-like SH-SY5Y cell line [30], further confirming the boosting effect of this neurotrophin on cholesterol biosynthesis in neurons [188]. On the other hand, BDNF significantly reduces the internalization of extracellular cholesterol and apoE interaction in the same neuronal cell line [30]. As already mentioned, the uptake of cholesterol is mediated by several receptors, including LDLr, VLDLr, apoER2 and LRP1 [255], widely expressed in the brain. The downregulation of these receptors is mediated by LXR-beta, a crucial regulator of cholesterol homeostasis in neurons, which increases following BDNF treatment: hence, BDNF reduces cholesterol uptake in neurons by raising LXR expression [30]. Collectively, these results highlight that BDNF orchestrates a complex homeostatic regulation, by enhancing the biosynthesis of cholesterol, and by suppressing its uptake in neurons. The reduction of extracellular cholesterol uptake in neurons may be attributable to the important neuroprotective role of BDNF. Indeed, if on one hand proper cholesterol levels are indispensable for assuring the correct induction of key neuronal processes, an excess of cholesterol in the brain can be toxic, causing apoptosis [256,257,258,259] and contributing to the formation and deposit of the beta-amyloid peptide [260]. 

The crucial role of neurotrophins in the regulation of cholesterol metabolism is further strengthened by few studies, which specifically investigated the involvement of the pan-neurotrophin receptor p75NTR. p75NTR appears to be a potent regulator of the enzymes involved in cholesterol biosynthesis in neuronal cultures and in various neuroblastoma cell lines. Notably, a relationship between p75NTR and cholesterologenic enzymes has been demonstrated by comparing p75NTR-positive and p75NTR-negative PC12 cells. These cells were treated with neocarzinostatin (NCS), an antimitotic agent capable of inducing cell death. Interestingly, the susceptibility to NCS was higher in p75NTR-positive cells than in p75NTR-negative cells. p75NTR expression was associated with an upregulation of enzymes responsible for cholesterol biosynthesis, such as HMGCR, farnesyl-diphosphate synthase, and 7-dehydro-cholesterol reductase. In order to understand whether the pro-apoptotic effects of p75NTR depended on increased cholesterol biogenesis, the authors used mevastatin to inhibit HMGCR. Upon mevastatin administration, NCS susceptibility between p75NTR-positive and p75NTR-negative cells was similar, indicating that cholesterol biosynthesis contributes to potentiate the pro-apoptotic effects of p75NTR [246]. The dependence of cholesterol biosynthesis on p75NTR activity is further sustained by Korade and colleagues. Even though the reduction in cholesterol biosynthesis in adult neurons is a widely accepted model, some neuron types may retain the capability to synthetize cholesterol in the adult brain, as the cholesterol biosynthetic enzymes HMGCR and DHCR7 are co-localized in some cortical, hippocampal and cholinergic neurons in the adult mouse brain. Considering that hippocampal and cholinergic neurons express p75NTR, the authors hypothesized that p75NTR could modulate the expression levels of enzymes belonging to the cholesterol biosynthetic pathway. Indeed, they silenced p75NTR expression in Neuro2a cells and in primary cerebellar neuronal cultures, and found that p75NTR suppression determined a significant decrease in the expression of HMGCR and DHCR7, possibly via the SREBPs-dependent pathway [249].

Successively, it has been found that the effects of p75NTR on the regulation of cholesterol and lipid biosynthetic genes are mediated by NRIF, both in vitro and *in vivo*. In fact, these findings imply that the NRIF-regulated endogenous cholesterol biosynthesis is crucial for neuronal homeostasis and that the addition of exogenous cholesterol cannot prevent the harmful effects derived from an altered biosynthesis [250]. 

Recently, it has been shown that p75NTR, in response to NGF or pro-NGF, is able to modulate the expression of LDLr in Huh7 hepatocytes, by inducing SREBP2 cleavage. Signal cascades leading to SREBP2 cleavage include the activation of p38 MAPK and caspase-3: p38 MAPK increases caspase-2 phosphorylation, thus reducing its interaction with caspase-3. Caspase-3, for its part, determines the cleavage of SREBP2, which exhibits a caspase-3 cleavage site. Then, SREBP2 induces the transcription of *ldlr* and other lipogenic genes. Thereby, the SREBP2 pathway mediated by the neurotrophin/p75NTR system differs from that induced by sterol deficiency, even though they could functionally interact under stress conditions and in case of abnormal lipid accumulation [247,248].

## 4. Conclusions

Even though initially identified as specific neurotrophic factors selectively committed to the survival and the differentiation of neuronal cells, compelling evidence highlights the crucial role of NTs in the regulation of cell metabolism in different cell contexts. The current knowledge summarized in this review underlines the crucial involvement of NTs in metabolic processes related to the control of glucose homeostasis, autophagy, oxidative stress, energy expenditure and lipid metabolism. Notably, some members belonging to the NT family also show important modulatory activities on cholesterol metabolism. When evaluated as a whole, literature data seem to suggest that, independently of the cell context, NGF and BDNF may promote an overall activation of cholesterol biosynthesis in different cell types [188,239,248]. Conversely, the effect on cholesterol uptake can be differently modulated in neuronal cells: specifically, BDNF suppresses cholesterol uptake [30], whereas NGF stimulates the internalization of lipoprotein particles [242]. This discrepancy can be explained by considering the different experimental setups, and the different roles potentially exerted by the two NTs. Indeed, cholesterol requirements are strongly increased to sustain neuritogenesis and synaptogenesis: thus, NGF may promote neuronal differentiation by enhancing LDL uptake to support the formation of new membranes. On the contrary, the reduction of cholesterol uptake induced by BDNF in already differentiated neuronal cells may represent a protective mechanism to prevent the toxicity induced by excessive cholesterol. Despite some hints on how NTs regulate cholesterol metabolism are now available, some information is still lacking, and a number of unanswered questions deserve further investigation. For instance, unlike BDNF, there are no systematic studies aimed at evaluating the prospective role of NGF in the regulation of cholesterol metabolism in astrocytic cells. Considering that neurons depend on cholesterol produced by astrocytes in the adult brain, it could be fruitful to comprehend whether NGF is able to affect the intercellular cholesterol transport, whose proper regulation is indispensable to guarantee brain physiology. Furthermore, the role of NTs other than NGF and BDNF, such as NT-3 and NT-4, is completely missing in the context of cholesterol homeostasis. A deeper comprehension of the molecular aspects linking NTs and cholesterol metabolism may be useful to better contextualize the involvement of NTs in different brain disorders and, more extensively, in a plethora of pathological conditions characterized by the loss of cholesterol homeostasis.

## Figures and Tables

**Table 1:** List of experimental evidence summarizing the role of neurotrophins in the modulation of cholesterol metabolism. ↑ indicate an increase; ↓ indicates a decrease.

Neurotrophin/Receptor	Cell Context	Effects	References
NGF	PC12 cell line	↑ HMGCR activity	[239]
NGF/TrkA	PC12 cell line	↑ apoER2 proteolytic processing ↑ apoE transcription	[244,245]
	GT1–1 Trk cell line; PC12 cell line	↑ LRP1 endocytic activity ↑ *LRP1* mRNA levels	[240,241]
NGF/TrkA pro-NGF/p75NTR pro-BDNF/p75NTR	PC6.3 cell line; Cultured septal neurons from embryonic rat brain	↑ LDLr expression ↑ Lipoprotein uptake	[242]
NGF/p75NTR	PC12 cell line	↑ Cholesterogenic enzymes	[246]
NGF/p75NTR pro-NGF/p75NTR	Huh7 hepatocyte cells	↑ Lipid- and cholesterol-associated proteins	[247,248]
BDNF/TrkB	Cerebral cortex; Hippocampal neurons from embryonic rats	↑ *hmgcr* and *mpd* transcripts	[188]
	NHA cells U87 MG cell line	↑ apoE biosynthesis; ↑ *ABCA1* levels; ↑ Cholesterol efflux;	[30]
	SH-SY5Y cell line	↑ *hmgcr* mRNA levels; ↓ Extracellular cholesterol uptake	[30]
p75NTR	Neuro2a cell line; Primary cerebellar neuronal cultures	↑ *hmgcr* and *dhcr7*	[249]
NRIF/p75NTR	Neuro2a cell line	↑ Cholesterogenic enzymes	[250]
